# Life-Threatening Cardiogenic Shock Related to Venlafaxine Poisoning—A Case Report with Metabolomic Approach

**DOI:** 10.3390/metabo13030353

**Published:** 2023-02-27

**Authors:** Romain Magny, Bruno Mégarbane, Pauline Guillaud, Lucie Chevillard, Nicolas Auzeil, Pauline Thiebot, Sebastian Voicu, Isabelle Malissin, Nicolas Deye, Laurence Labat, Pascal Houzé

**Affiliations:** 1 Laboratoire de Toxicologie, Fédération de Toxicologie, AH-HP, Hôpital Lariboisière, 75010 Paris, France; 2 Inserm, UMRS-1144, Université Paris Cité, 75006 Paris, France; 3 Réanimation Médicale et Toxicologique, Fédération de Toxicologie de l’AH-HP, Hôpital Lariboisière, 75010 Paris, France; 4 Université Paris Cité, CNRS, CiTCoM, 75006 Paris, France; 5 Université Paris Cité, Faculté de Sciences Pharmaceutiques et Biologique, Unité de Technologies Chimiques et Biologiques pour la Santé (UTCBS), CNRS UMR8258, Inserm UMR-8258, 75006 Paris, France

**Keywords:** metabolomics, molecular network, analytical toxicology, clinical toxicology, venlafaxine, poisoning, ECMO, management, cardiotoxicity

## Abstract

Metabolomics in clinical toxicology aim at reliably identifying and semi-quantifying a broad array of endogenous and exogenous metabolites using dedicated analytical methods. Here, we developed a three-step-based workflow to investigate the metabolic impact of the antidepressant drug venlafaxine in a poisoned patient who developed life-threatening cardiac failure managed with extracorporeal membrane oxygenation. Both targeted quantitative and untargeted semi-quantitative metabolomic analyses using liquid chromatography hyphenated to high-resolution tandem mass spectrometry were performed to determine the plasma kinetics of venlafaxine, *O*-desmethyl-venlafaxine, and *N*-desmethyl-venlafaxine and to identify sixteen different venlafaxine-derived metabolites including one unknown (*i.e.*, venlafaxine conjugated to a hexosyl-radical), respectively. Correlations between the quantitative metabolomic data and annotated endogenous metabolites suggested impaired amino acid and lipid metabolism, Krebs cycle, and kynurenine pathway. This preliminary study represents a first step towards a more extensive application of toxicometabolomics in clinical toxicology and a useful workflow to identify the biomarkers of toxicity.

## 1. Introduction

Depression affects more than 300 million people worldwide [1,2,3,4]. Venlafaxine, a serotonin and norepinephrine reuptake inhibitor, is a first-line treatment of depressive episodes, anxiety disorders, and phobias [5,6,7]. Liver venlafaxine metabolism consists of demethylation mainly to *O*-desmethyl-venlafaxine by cytochrome P450 (CYP) 2D6 and, to a lesser extent, to *N*-desmethyl-venlafaxine by CYP3A4 [8,9,10]. Demethylated metabolites are then glucurono- and sulfo-conjugated before excretion in urine, which represents the major route of venlafaxine elimination [8,9,10]. Venlafaxine and its active *O*-desmethyl-venlafaxine metabolite act by potentiating monoamines in the central nervous system including serotonin, norepinephrine and, to a lesser extent, dopamine [8,9,11].

A venlafaxine overdose may result in encephalopathy, consciousness impairment, seizures, metabolic disturbances, rhabdomyolysis, and serotoninergic syndrome. Life-threatening cardiotoxicity may occur from sodium channel blockage (membrane stabilizing activity), leading to QT prolongation and QRS widening on the electrocardiogram, ventricular arrhythmia, and cardiac failure [12,13,14,15]. Besides gastrointestinal decontamination if indicated, its management relies on mechanical ventilation, 8.4% sodium bicarbonate, inotropic drugs, and vasopressors, and may require veno-arterial extracorporeal membrane oxygenation (ECMO) in the rare cases of refractory cardiac failure or arrest [16,17,18,19,20].

Metabolomics using liquid chromatography hyphenated to high-resolution mass spectrometry (LC-HRMS) have been suggested as an emerging approach to clarify the mechanisms of toxicity and identify prognostic biomarkers [21,22,23,24,25]. This strategy relies on targeted analyses quantifying well-known molecules [21,22] and additionally involves challenging untargeted analyses to detect and quantify hundreds of other metabolites [21,22]. Recently, the molecular network (MN) was proposed as a reliable data processing strategy to facilitate the identification of a broad array of diverse molecules including endogenous and exogenous metabolites [26,27,28,29,30]. Whereas the gold standard for quantification involves a multi-point calibration curve using internal standards [31], whereby availability may be limited in real life, the semi-quantification of metabolites identified based on such MNs is possible even though its reliability and accuracy for quantification has to be assessed.

Metabolomic studies performed on rodent models to investigate the mechanisms of venlafaxine action showed specific changes in endogenous metabolites associated with cell death, impaired lipid and amino acid metabolisms, and/or altered energy production [32,33]. Both the Krebs cycle and fatty acid metabolism were targeted by venlafaxine in a time- and dose-dependent manner. These studies provided new insights into the mechanisms involved in venlafaxine-related antidepressant activity. However, studies are still needed to clarify whether these mechanisms reported in rodents are involved in venlafaxine-related toxicity in humans.

In this study, we thus investigated the metabolic impacts of venlafaxine in a severely venlafaxine-poisoned patient who presented a cardiogenic shock requiring ECMO. For this purpose, we have developed an original toxicometabolomics workflow through a three-step approach. The first step deals with a targeted analysis regarding the absolute quantification of venlafaxine and two of its demethylated metabolites, namely *O*-desmethyl-venlafaxine and *N*-desmethyl-venlafaxine, allowing one to access several pharmacokinetic parameters. A second step involved a non-targeted approach using LC-HRMS, allowing for an exhaustive annotation and semi-quantification of numerous metabolites of venlafaxine, a prior step to establish their kinetic profile. The third step corresponds to the annotation and semi-quantification of endogenous metabolites in plasma extracts. Finally, we assessed plasma level correlations between venlafaxine and endogenous plasma metabolites to investigate the plasma metabolome alteration induced by this drug intake during severe intoxication.

## 2. Case Report

**Figure 1:**
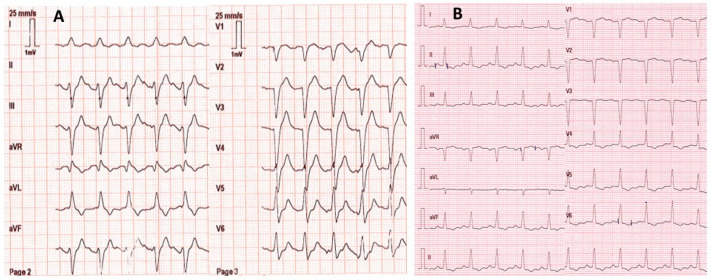
Electrocardiograms on the scene ~20 h post-venlafaxine ingestion (**A**) and on intensive care unit admission after mechanical ventilation, fluid, 8.4% sodium bicarbonate (750 mL), and norepinephrine infusion (**B**).

A 53-year-old female with no remarkable medical history except for depression was found by firefighters at home comatose (Glasgow coma score: 3 with mydriasis), hypotensive (blood pressure: 80/55 mmHg; heart rate: 123/min), and in respiratory distress (respiratory rate: 18/min; SpO2: 56% on room air). She had presumably ingested an unknown dose of venlafaxine and oxazepam ~20 h before examination. On the scene, electrocardiogram showed sinus tachycardia with an enlarged QRS complex (0.160 s) but normal corrected QT interval (0.384 s) (Figure 1). The patient was rapidly intubated and mechanically ventilated. She received fluids (2 L 0.9% NaCl) and 8.4% sodium bicarbonate (750 mL). Norepinephrine infusion was started. She was transported to the intensive care unit (ICU). Upon admission, electrocardiogram showed normal QRS complexes (duration: 0.08 s). Laboratory tests showed arterial pH: 7.39; PaO_2_/FiO_2_: 264 mmHg; blood lactate: 4.3 mmol/L; serum creatinine: 113 µmol/L; prothrombin time ratio: 88%; and serum aspartate/alanine aminotransferases: 110/31 IU/L. Chest X-ray showed right three-lobe aspiration pneumonia. Echocardiography showed a marked decrease in left ventricular ejection fraction (LVEF) to less than 20% without segmental wall motion abnormalities. High-sensitivity cardiac troponin I was 2522 ng/L (chemiluminescent microparticle immunoassay (CMIA), Abbott Alinity^®^; N < 16) and serum brain natriuretic peptide was 2470 ng/L (CMIA, Abbott Alinity^®^; decisional threshold: 100). Routine toxicological screening, using high-performance liquid chromatography coupled to ultraviolet spectrophotometry and mass spectrometry detection, was negative except for benzodiazepines. The plasma acetaminophen concentration was 23.8 mg/L. The plasma venlafaxine concentration was estimated to be 9043 ng/mL (therapeutic range: 100–400) using gas chromatography coupled to a nitrogen–phosphorus detector. The plasma oxazepam and nordiazepam concentrations were estimated to be 4786 ng/mL (therapeutic range: 200–1500) and 86 ng/mL (120–800), respectively.

Initial management of cardiovascular failure included dobutamine (up to 20 µg/kg/min), norepinephrine (up to 3.1 µg/kg/min), and epinephrine infusions (up to 1.4 µg/kg/min) to maintain blood pressure. However, two hours later, no improvement in cardiac (LVEF < 10%) and organ functions (blood lactate: 13.0 mmol/L; PaO_2_/FiO_2_: 152 mmHg; oliguria with serum creatinine: 144 µmol/L; prothrombin time ratio: 40%; and aspartate/alanine aminotransferases: 2859/1990 IU/L) was observed. Due to refractoriness of the presumed drug-induced cardiac failure with rapidly worsening multiorgan failure, veno-arterial ECMO was implemented via femoral vessel cannulation at the bedside. The patient received activated charcoal (50 g), *N*-acetylcysteine (300 mg/kg/day), hydrocortisone succinate (200 mg/day), cefotaxime (4 g/day), and metronidazole (1.5 g/day). Continuous veno-venous hemodialysis was required due to acute kidney injury with anuria. The clinical course in the ICU was complicated by *Enterobacter cloacae*-related ventilation-acquired pneumonia treated with cefepime and several hemorrhage episodes at the cannulation site requiring red blood cell, platelet, and fresh plasma transfusion. Organ functions progressively improved (peak blood lactate: 29 mmol/L; peak serum creatinine: 493 µmol/L; and peak aspartate/alanine aminotransferases: 6451/2994 IU/L on Day 1), allowing the progressive weaning of vasopressors (Day 4), ECMO (Day 5), mechanical ventilation (Day 11), and intermittent hemodialysis (Day 14). The patient was discharged to the medical (Day 25) and then to the psychiatric ward (Day 31) without sequelae.

## 3. Materials and Methods

### 3.1. Chemical and Reagents

Water, *tert*-butyl-methyl-ether (MTBE), acetonitrile (ACN), isopropanol (IPA), and methanol (MeOH) of LC-MS grade were obtained from Sigma-Aldrich (Saint-Quentin-Fallavier, France). Venlafaxine, *O*-desmethyl-venlafaxine, *N*-desmethyl-venlafaxine, venlafaxine-d6, buprenorphine-d4, and THC-COOH-d3 were purchased from LoGiCal^®^ (LGC GmBH, Molsheim, France).

### 3.2. Quantitative Targeted Analyses

Quantitative targeted analyses were performed using a method validated according to the EMA guideline, including repeatability and intermediate precision lower than 15% for quality control.

Sample preparation: The calibration samples were prepared using blank plasma spiked with a solution of certified venlafaxine, *O*-desmethyl-, and *N*-desmethylvenlafaxine. The calibration curve of each quantified compound ranged between 20 ng/mL and 5000 ng/mL. The calibration samples (20, 50, 100, 500, 1000, 2000, and 5000 ng/mL) and the quality control (30, 750, and 3000 ng/mL) were freshly prepared on the day of analysis. For preparation, liquid–liquid extraction was performed from each 100 µL sample diluted in 500 µL carbonate buffer (0.1 M, pH = 10). A volume of 200 µL of the internal standard (i.e., venlafaxine-d6 diluted in ACN, 10 ng/mL) was spiked in each sample. The sample was then vortexed for 10 min and extracted twice with 1.8 mL MTBE. Following the centrifugation step, the organic layers were evaporated under gentle steam of nitrogen at 35 °C and dry extracts were dissolved in H_2_O/MeOH (50/50, *v*/*v*). A volume of 10 µL was injected into the LC-HRMS analytical system.

Analytical conditions: The LC-HRMS system was based on the Vanquish^®^ LC pump and autosampler coupled to a QExactive Focus^®^ mass spectrometer equipped with a heated electrospray ionization (HESI) probe operating in polarity switching mode (ThermoFisher Scientific, Bremen, Germany). The analytical system was managed using TraceFinder^®^ 4.0 software (ThermoFisher Scientific). LC was performed on an Accucore^®^ Phenyl Hexyl (100 × 2.1 mm, 2.6 µm (ThermoFisher Scientific)) column maintained at 40 °C. The flow rate was set at 500 µL/min. A binary gradient system was used for the elution and consisted of water with 2 mmol/L formate ammonium as solvent A and a mixture of methanol/acetonitrile with 2 mmol/L formate ammonium (50:50) as solvent B. Both solvents A and B also contained 0.1% formic acid. Eluent was maintained at 1% B for 1 min, increased to 99% B in 10 min, held at 99% B for 1.5 min before returning to 1% B, and finally was held for 4 min. The mass spectrometer parameters were as follows: ionization voltage was 3.0 kV for positive ion mode and 2.5 kV for negative ion mode; sheath gas and auxiliary gas were 35 and 15 arbitrary units, respectively; S-lens RF level was 60; and vaporizer temperature and capillary temperature were both set at 320 °C. Nitrogen was used for spray stabilization, for collision-induced dissociation experiments in the HCD cell, and as the damping gas in the C-trap. The automatic gain control target was fixed to 1E5 for MS/MS experiments. Transient time was fixed to 120 and 50 ms for full MS and MS/MS scans, respectively. The mass spectrometer was calibrated in the positive and negative modes twice weekly prior to analyses. Data were acquired in full-scan in data-dependent MS2 (ddMS2) mode. In this mode, a full-scan acquisition was performed at a resolution of 70,000 (at *m*/*z* 200) between *m*/*z* 100 and 1000, followed by the acquisition of MS/MS spectra, at a resolution of 17,500 with an isolation window of *m*/*z* 2. The acquisition of MS/MS spectra was based on a list of ~1500 compounds including venlafaxine, *O*-desmethyl-venlafaxine, *N*-desmethyl-venlafaxine, and the internal standard venlafaxine d6. Acquisition of MS and MS/MS data was managed using Thermo Scientific TraceFinder^®^ software.

Data processing: for the targeted quantitative analyses, the data processing step was performed using TraceFinder^®^ software to generate the calibration curve and to quantify venlafaxine, *O*-desmethyl-venlafaxine, and *N*-desmethyl-venlafaxine.

Pharmacokinetic analyses: The plasma concentration–time profile of venlafaxine and its two main metabolites (*O*-desmethyl-venlafaxine and *N*-desmethyl-venlafaxine) were analyzed using a standard non-compartmental method (Phoenix WinNonlin^®^ software version 8.3, Certara USA, Inc., Princeton, NJ, USA). The peak plasma concentration (C_max_) and time to peak plasma concentration (T_max_) for each compound were determined directly from measured data. The area under the plasma concentration–time curve (AUC_last_) was calculated using measured data points from the time of administration to the time of the last quantifiable concentration (C_last_) via a linear trapezoidal rule. The elimination half-life (t_1⁄2_) was calculated as t_1⁄2_ = Ln2⁄ λz, where λz was estimated as the slope of the log-linear terminal portion of the plasma concentration vs. time curve.

### 3.3. Non Targeted Analyses

Sample preparation: For the non-targeted analyses, a volume of 150 µL of LC-MS grade water, 350 µL of acetonitrile, containing the internal standards buprenorphine-d4 and THC-COOH-d3, and 350 µL of isopropanol were added to 100 µL of plasma samples. Samples were quickly vortexed and then kept at −20 °C for one hour to precipitate proteins. The precipitated proteins were pelleted via centrifugation at 14,000 rpm for 10 min. Supernatants were collected and solvents were evaporated at 40 °C under gentle steam of nitrogen. Dry residues were dissolved in H_2_O/MeOH (50/50, *v*/*v*). Prior to the injection, quality controls (QCs) were prepared and consisted of an equivolumetric mixture of each plasma extract. A volume of 10 µL of the extracts was injected into the analytical system. QCs were injected periodically throughout the analysis of all plasma extracts.

Analytical conditions: The analytical platform used for non-targeted analyses was based on the same instrument and analytical conditions as for the quantitative targeted analyses. Nevertheless, the acquisition of mass spectra and tandem mass spectra was performed in discovery data-dependent analysis mode. In this mode of acquisition, a full-scan acquisition was performed at a resolution of 70,000 between *m*/*z* 100 and 1000, followed by the acquisition of three MS/MS spectra on the three most intense ions, at a resolution of 17,500 with an isolation window of *m*/*z* 2. The dynamic exclusion mode used was set at 3 s for each selected ion.

Data processing: Raw data files acquired in positive and negative ion modes were converted into open-source mzXML files using MSConvert 3.0™ [34,35]. Data processing was performed using MZmine 2.53 as previously described [36,37]. Briefly, MS and MS/MS spectral data were extracted using a mass detection noise level set at 1E5 and 5E3, respectively. Then, extracted ion chromatograms were systematically built using the ADAP algorithms (minimum group size of 4 scans, a group intensity threshold of 500,000, and an *m*/*z* tolerance of 10 ppm) [38]. The ADAP wavelets chromatogram deconvolution algorithm was then applied and set at the following parameters: signal-to-noise ratio: 6; coefficient/area ratio: 40; peak duration range: 0.05–1.5 min; and retention time wavelet range: 0.0–0.15 min. De-isotope chromatograms were grouped using the isotopic peaks grouper algorithm set at *m*/*z* and t_R_ tolerances of 10 ppm and 0.1 min, respectively. The peak alignment of samples was then achieved using the join aligner method with parameters set at *m*/*z* and t_R_ tolerances of 10 ppm and 0.15 min, respectively. MS/MS scans were associated with the corresponding MS scans using an *m*/*z* and t_R_ tolerance of 10 ppm and 0.15 min, respectively. The gap-filling process was performed on the peak list using the so-called module “same RT and *m*/*z* range gap filler” with *m*/*z* tolerance of 10 ppm. These data processing steps led to a matrix listing the peak intensities for each analyzed sample associated with a unique *m*/*z* and retention time value. It also generated a file (.mgf format) containing the MS/MS spectra data, allowing us to generate the molecular network.

Molecular network generation: The MNs were generated using the feature-based molecular networking workflow of the Global Natural Products Social platform and using MetGem software [26,39]. MNs were built using LC-MS/MS data obtained together from all analyzed plasma samples. The following settings were used to build the network: minimum pairs Cos: >0.60; parent ion mass tolerance: 0.02 Da; fragment ion mass tolerance: 0.02; network topK: <150; minimum matched peaks: 4; and minimum cluster size: 2. The library spectra inquiries were performed using the same parameter values as those defined for the network building. The MNs were finally visualized and annotated using Cytoscape 3.4.0™ software (San Diego, CA, USA) [40].

Identification of exogenous and endogenous metabolites: The dereplication step of endogenous and exogenous metabolites was obtained using the GNPS database library and an in-house database. Furthermore, the structure assignment of venlafaxine and its metabolites as well as endogenous plasma metabolites was based on MS and MS/MS data, using a tolerance window of 5 and 15 ppm, respectively. Annotations were supported by experimental chromatographic retention time (t_R_) values. SIRIUS 5.4 was used to support molecular formula determination and metabolite identification [41].

Semi-quantification: The semi-quantification was based on the use of internal standard mixture spiked in plasma sample during the sample preparation. Intensities of each annotated metabolite were normalized individually to the internal standard intensity. Buprenorphine-d4 and THC-COOH-d3 were used as internal standards for positive and negation ion modes, respectively. Following the normalization step, annotated metabolites were filtered based on the coefficient of variation (CV%) of the QC. Only the annotated metabolites exhibiting a CV% value within the QC samples lower than 25% were considered for the rest of the analysis.

### 3.4. Statistical Analyses

Pearson correlation analyses were performed using GraphPad Prism 9 software^®^ (GraphPad Software, La Jolla, CA, USA) with a risk set at 0.05 (* *p* < 0.05, ** *p* < 0.01, and *** *p* < 0.001).

## 4. Results

### 4.1. Pharmacokinetics of Venlafaxine, O-desmethyl-venlafaxine, and N-desmethyl-venlafaxine Metabolites

**Figure 2:**
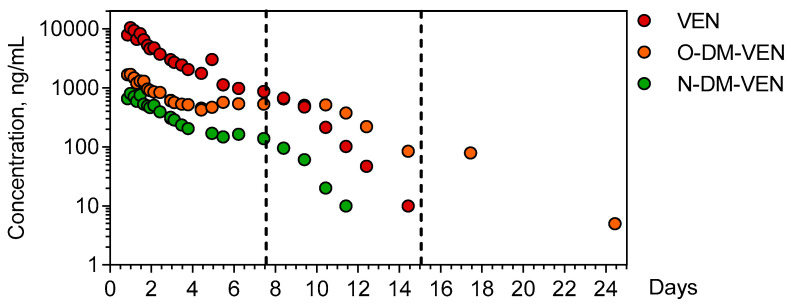
Time-course of plasma venlafaxine, *O*-desmethyl venlafaxine, and *N*-desmethyl-venlafaxine. The dotted lines indicate the end of important therapies used to manage the patient (extracorporeal membrane oxygenation + continuous veno-venous hemodialysis for the first line and intermittent hemodialysis for the second line). VEN = Venlafaxine, O-DM-VEN = *O*-desmethyl-venlafaxine, and N-DM-VEN = *N*-desmethyl-venlafaxine.

Venlafaxine and its demethylated metabolites *O*-desmethyl-venlafaxine and *N*-desmethyl-venlafaxine were quantitatively determined in the 26 available plasma samples of the patient over a 25-day period (Figure 2).

The ingestion time was presumed but the ingested venlafaxine amount could not be determined. The first plasma sample obtained 20 h post-ingestion contained 9515 ng/mL venlafaxine (therapeutic range: 100–400), 1662 ng/mL *O*-desmethyl-venlafaxine (100–400), and 653 ng/mL *N*-desmethyl-venlafaxine. C_max_ of venlafaxine (9735 ng/mL) was achieved 23.67 h post-ingestion. The estimated elimination half-life of venlafaxine was 20.6 h (λz = 0.034 h^−1^), with an AUC_last_ of 66,7299 ng.h/mL. The C_max_ of *O*-desmethyl-venlafaxine and *N*-desmethyl-venlafaxine (1910 and 770 ng/mL) was obtained at 20.5 and 23.67 h, respectively. Metabolic ratios were 0.3 and 0.06 for *O*-desmethyl-venlafaxine and *N*-desmethyl metabolites, respectively.

### 4.2. Identification of Phase-I and Phase-II Metabolites of Venlafaxine

**Figure 3:**
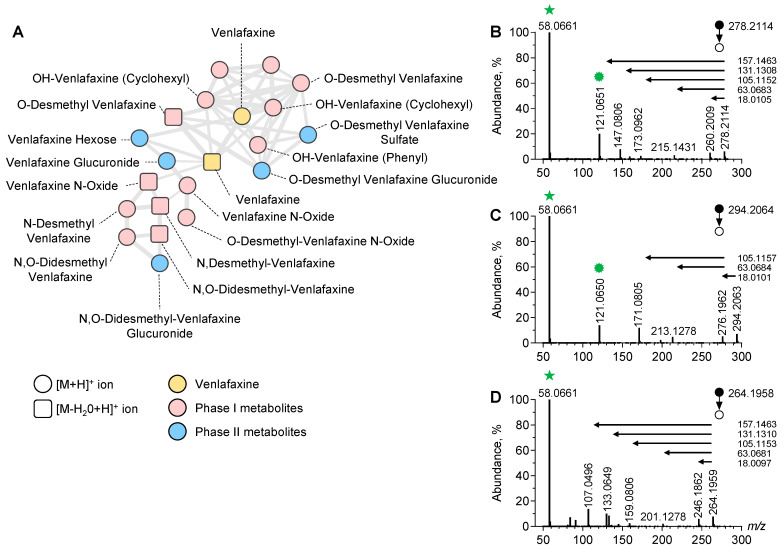
Molecular network of venlafaxine and metabolites generated in positive ion mode. Focus on the cluster containing venlafaxine and its corresponding metabolites (**A**). MS/MS spectra of the [M+H]^+^ ion of venlafaxine (**B**), OH-venlafaxine (**C**), and *O*-desmethyl-venlafaxine (**D**) displaying product ions and neutral losses involved in networking.

To identify the additional metabolites of venlafaxine, we used an untargeted approach based on LC-ESI-HRMS/MS analysis of plasma sample extracts prior to data processing and MN implementation (Figure 3).

In the positive and negative ion modes, a typical chromatogram showed elution of amino acids and tricarboxylic acids before 3 min, venlafaxine and metabolites at 3–5 min, and lipids after 8 min (Supplementary Figure S1). The generated MN displayed clusters including one referring to structurally venlafaxine-related compounds. This cluster contained 20 nodes including one corresponding to the [M+H]^+^ ion of venlafaxine. The MS/MS spectrum of the [M+H]^+^ ion of venlafaxine at *m*/*z* 278.2114 displayed a product ion at *m*/*z* 260.2009 corresponding to a loss in H_2_O from the cyclohexyl moiety of venlafaxine. Additional product ions at *m*/*z* 215.1431, *m*/*z* 173.0962, and *m*/*z* 147.0806 corresponded to the sequential loss in the dimethylamine radical and fragmentation of the dehydrated cyclohexyl moiety of venlafaxine. Finally, a product ion at *m*/*z* 121.0650 corresponded to the methoxyphenyl radical of venlafaxine, while the ions at *m*/*z* 58.0661 were assigned to the methyl-aziridinium ion. A proposed fragmentation pattern of venlafaxine is presented in Supplementary Figure S2. Ultimately, the MS/MS venlafaxine spectrum revealed a set of diagnostic ions representing footprints of hydroxycyclohexyl, methoxyphenyl, and dimethylamine radicals, suggesting that the networking of the venlafaxine cluster can be useful for identifying venlafaxine metabolites.

Manual inspection of the nodes present in the venlafaxine cluster allowed for the identification of phase-I and II metabolites including *O*-desmethyl-venlafaxine and *N*-desmethyl-venlafaxine (Figure 3). Of note, several nodes were attributed in this cluster to hydroxylated venlafaxine metabolites, which are structural isomers since enzymatic hydroxylation can occur in Positions 2, 3, 5, and 6 of 4-methoxyphenyl and on the cyclohexyl ring. However, despite the same *m*/*z* value at 296.2064, hydroxylated venlafaxine metabolites displayed different retention times, depending on the hydroxylation site and resulting in multi-nodes in the MN related to structural isomers (Supplementary Figure S3). The manual inspection of MS/MS spectra allowed for identifying whether hydroxylation occurred in the cyclohexyl or methoxyphenyl radical. MS/MS spectra displayed a peak at *m*/*z* 294.2063 corresponding to the [M+H]^+^ ion of an OH-venlafaxine metabolite and a peak at *m*/*z* 213.1278 suggesting a loss in the two hydroxylated functions in the cyclohexyl radical and dimethylamine moiety (Figure 3C). On the same MS/MS spectra, the product ion at *m*/*z* 121.0650 allowed for the identification of the methoxyphenyl radical of venlafaxine. Based on the MS/MS spectra inspection, the peak detected at t_R_ of 2.70 min in the ion chromatogram at *m*/*z* 294.2064 was identified as the hydroxylation of the cyclohexyl radical of venlafaxine.

Sixteen venlafaxine metabolites have been annotated using LC-HRMS data acquired in both ESI positive and negative ion modes, including six phase-I and ten phase-II metabolites. The analytical characteristics of these metabolites including the retention times and the exact mass of parent and diagnostic product ions are listed in Supplementary Table S1. For each metabolite, the annotation level was reported according to standard metabolomic initiative guidelines. Phase-II metabolites were also detected in the negative ion mode and corresponding MN. A proposed metabolic network including the additional venlafaxine metabolites is presented in Supplementary Figure S4.

**Figure 4:**
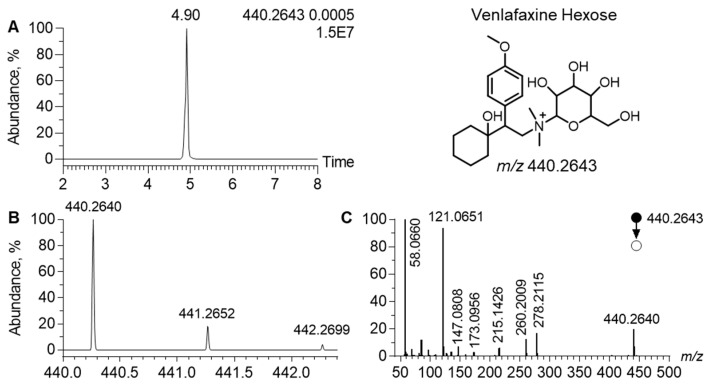
Analytical features of the putatively annotated glycosylated venlafaxine metabolite. Extracted ion chromatogram at *m*/*z* 440.2643 corresponding to the [M+H]^+^ ion of venlafaxine-hexose (**A**). Mass spectra of venlafaxine-hexose and its corresponding isotopic pattern (**B**). MS/MS spectra of the [M+H]^+^ ion of the putatively annotated venlafaxine-hexose (**C**).

In the MN generated in the positive ion mode, the cluster of venlafaxine displayed a node, corresponding to a precursor ion at *m*/*z* 440.2642, linked to venlafaxine and venlafaxine glucuronide (Figure 4). In the related MS/MS spectra, a product ion was observed at *m*/*z* 278.2115. It corresponded to the [M+H]^+^ ion of venlafaxine (Δppm, 1), resulting from the neutral loss of 162.0525, attributed to a hexosyl radical (Δppm, 2). Such a neutral loss is known to be diagnostic of the glycosyl radical. Regarding the chromatographic data, the extracted ion chromatogram of *m*/*z* 440.2642 led to a peak at t_R_ of 4.90 min. Analytical characteristics strongly suggested that *m*/*z* 440.2642 corresponded to venlafaxine hexose. The difference between the theoretical and experimental *m*/*z* values of the [M+H]^+^ ion of venlafaxine hexose was lower than 1 ppm and the isotopic pattern was consistent with its molecular formula, i.e., C_23_H_37_NO_7_. MS/MS spectra additionally exhibited all diagnostic venlafaxine ions (i.e., the hydroxycylohexyl, methoxyphenyl, and dimethylamine radicals). However, analytical data could not readily determine the stereochemistry of the hexosyl radical.

### 4.3. Comparison of the Absolute Quantification and Semi-Quantification of Venlafaxine, O-Desmethyl-Venlafaxine, and N-Desmethyl-Venlafaxine

**Figure 5:**
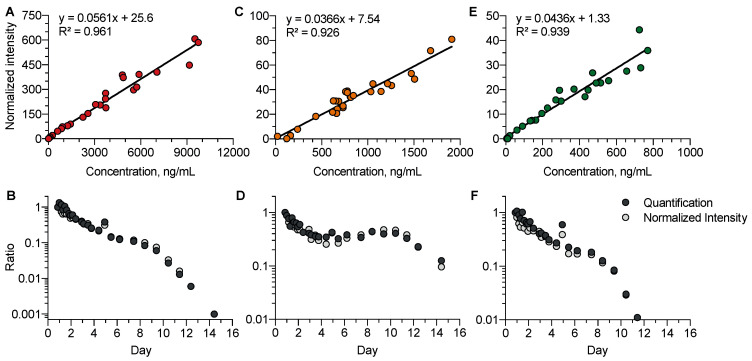
Comparison between the quantitative and semi-quantitative measurements of plasma venlafaxine, *O*-desmethyl-venlafaxine, and *N*-desmethyl-venlafaxine concentrations. Correlations between the absolute concentrations and normalized intensities of venlafaxine (**A**), *O*-desmethyl-venlafaxine (**C**), and *N*-desmethyl-venlafaxine (**E**). Comparisons of the kinetic profiles obtained with quantified and semi-quantified data for venlafaxine (**B**), *O*-desmethyl-venlafaxine (**D**), and *N*-desmethyl-venlafaxine (**F**). Quantitative and semi-quantitative data were reported at the first time-point of the kinetics.

We investigated whether, in the absence of available analytical standards, the so-called normalized intensity strategy for semi-quantification could be relevant to study the kinetic profiles of venlafaxine metabolites (Figure 5). Data accuracy using normalized intensities and absolute quantification was studied using a correlation approach for venlafaxine (R^2^ = 0.97), *O*-desmethyl-venlafaxine (R^2^ = 0.92), and *N*-desmethyl-venlafaxine (R^2^ = 0.94). The strong correlation covered a high dynamic range of three to four logarithmic units. The normalized intensity strategy appeared to be suitable for investigating the kinetic profiles of venlafaxine metabolites including five phase-I and seven phase-II metabolites, running out of analytical standards.

### 4.4. Kinetic Profiles of the Semi-Quantified Venlafaxine Metabolites

**Figure 6:**
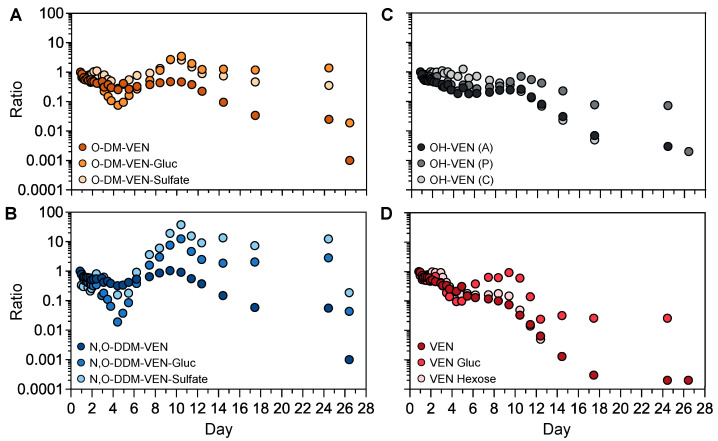
Kinetic profiles of phase-I and phase-II metabolites of venlafaxine based on semi-quantitative data. Time-course profiles for *O*-desmethyl-venlafaxine and its glucurono- and sulfo-conjugated molecules (**A**), *N*,*O*-didesmethyl-venlafaxine and its glucurono- and sulfo-conjugated derivatives (**B**), venlafaxine and its glucurono- and glycol-conjugated molecules (**C**), and isomers of hydroxylated venlafaxine (**D**). Results are expressed as the ratio to the first investigated plasma sample for each metabolite at each sampling time in order to compare metabolic profiles within the time between produced metabolites.

The semi-quantification method was applied to investigate the kinetic profiles of venlafaxine metabolites without available analytical standards (Figure 6). We first focused on the phase-II metabolites of *O*-desmethyl-venlafaxine. Taking into account that the first sample was obtained ~20 h post-ingestion, both plasma concentrations of *O*-desmethyl-venlafaxine-glucuronide and *O*-desmethyl-venlafaxine-sulfate slightly decreased during the first four days and then sharply rose between Days 5 and 10 before peaking on Day 10 (Figure 6A). Similar profiles were observed with phase-II *N*,*O*-didesmethyl-venlafaxine metabolites (i.e., *N*,*O*-didesmethyl-venlafaxine glucuronide and *N*,*O*-didesmethyl-venlafaxine-sulfate). All glucurono- and sulfo-conjugated metabolites of *O*-desmethyl-venlafaxine and *N*,*O*-didesmethyl-venlafaxine were detected in a window of 25 days (Figure 6B). Of note, the kinetic profiles of hydroxylated venlafaxine isomers were similar to those of *O*-desmethyl-venlafaxine (Figure 6C). Concentrations of venlafaxine glucose and venlafaxine hexose, two conjugated venlafaxine metabolites without a prior phase-I reaction, regularly decreased during 10 days, paralleling venlafaxine kinetics (Figure 6D).

### 4.5. Effects of Venlafaxine on Plasma Endogenous Metabolites

Besides venlafaxine and its sixteen identified metabolites, untargeted analysis data allowed for the detection of 214 various endogenous metabolites including amino acids, dipeptides, organic acids, organic amines, vitamins, and lipids. The annotation step was performed by querying in-house and GNPS database libraries. The proposed metabolite annotation was manually inspected and validated. Correlations between plasma concentrations of annotated metabolites and venlafaxine were negative for 77 compounds and positive for 50 other compounds (Supplementary Table S2).

**Figure 7:**
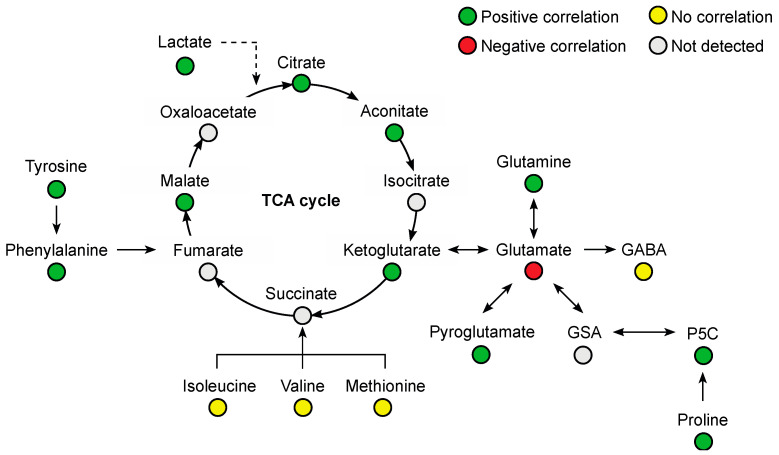
Summary of correlated metabolites belonging to the amino acid and Krebs (TCA) cycle pathways with plasma venlafaxine concentrations. Pearson correlation analyses were performed with a significance threshold set at 0.05.

Several amino acids were positively correlated with plasma venlafaxine, including phenylalanine (r = 0.76, *p* < 0.0001), glutamine (r = 0.75, *p* < 0.0001), tyrosine (r = 0.65, *p* = 0.0003), proline (r = 0.53, *p* = 0.0043), and tryptophan (r = 0.50, *p* = 0.0079) (Figure 7). Glutamic acid was the only amino acid that was negatively correlated (r = −0.57, *p* = 0.002). Regarding organic acids, a positive correlation was observed with malic acid (r = 0.77, *p* < 0.0001), citric acid (r = 0.65, *p* = 0.0002), aconitic acid (r = 0.64, *p* = 0.0003), and oxoglutaric acid (r = 0.45, *p* = 0.018).

**Figure 8:**
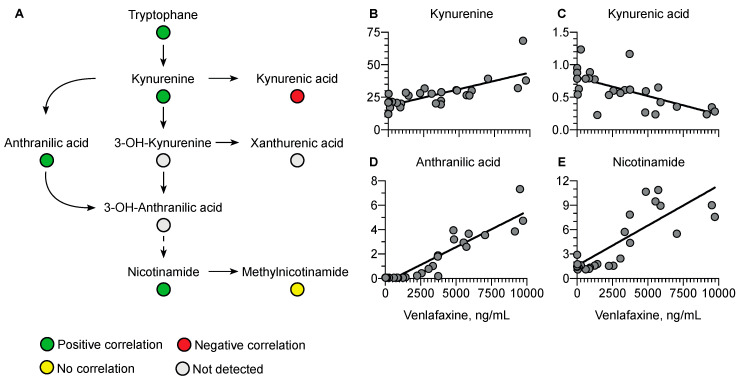
Summary of correlated metabolites belonging to the kynurenine pathway with plasma venlafaxine concentrations. Pearson correlation analyses were performed with a significance threshold set at 0.05.

Regarding the kynurenine pathway, a positive correlation was observed with kynurenine (r = 0.76, *p* < 0.0001), anthranilic acid (r = 0.92, *p* < 0.0001), and nicotinamide (r = 0.64, *p* = 0.0003) versus a negative correlation with kynurenic acid (r = −0.64, *p* = 0.0003) (Figure 8).

**Figure 9:**
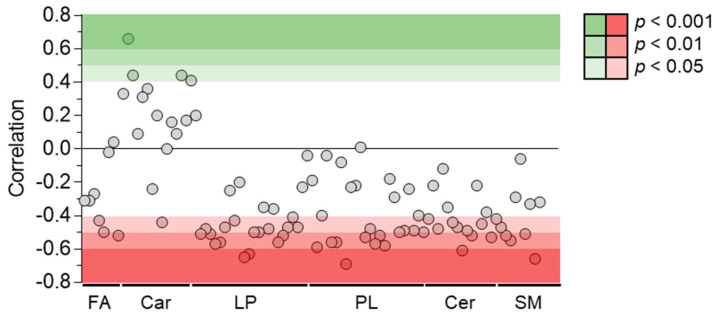
Pearson correlations between quantitative data of venlafaxine and lipid species. Pearson correlation analyses were performed with a significance threshold set at 0.05. FA = fatty acid, Car = Carnitine, LP = Lysophospholipid, PL = Phospholipid, Cer = Ceramide, and SM = Sphingomyelin.

Among the 214 identified compounds, 100 were lipids, especially of the phospholipid and sphingolipid species. Fifty-two lipid metabolites were correlated negatively with plasma venlafaxine, including forty phospholipids, eight sphingolipids, and three fatty acids (Figure 9). Phosphatidylcholine (PC) (16:0/20:5) regularly increased up to five times its initial plasma concentration. Correlations did not depend on the polar head of phospholipid since no significant differences between phosphatidylcholine, phosphatidylethanolamine, and phosphatidylinositol classes were observed. No correlation could be found between plasma venlafaxine and lipids in relation to the fatty acid (Supplementary Table S2). Regarding sphingolipids, about half of the annotated ceramide and sphingomyelin species showed a negative correlation with plasma venlafaxine.

## 5. Discussion

Although venlafaxine, a serotonin and norepinephrine reuptake inhibitor, is used worldwide as a first-line antidepressant drug [2,9], severe toxicity may occur in overdoses including cardiogenic shock [16,17,18,19,20]. Combining targeted and untargeted LC-HRMS analyses, we have reported an extensive metabolomics investigation in a life-threatening venlafaxine poisoning case.

Using a targeted approach, we first performed an absolute quantification of venlafaxine and its *O*-desmethyl and *N*-desmethyl metabolites in plasma, allowing for the determination of the pharmacokinetic parameters. Our assay allowed covering the whole venlafaxine concentration range reached in this poisoning case (from 20 to ~10,000 ng/mL), knowing that the toxic and fatal venlafaxine + *O*-desmethyl-venlafaxine concentrations are considered at 1000 ng/mL and 3000 ng/mL, respectively [42]. At the recommended 75–450 mg/day doses, venlafaxine follows linear pharmacokinetics with a volume of distribution of 7.5 ± 3.7 L/kg, an apparent plasma clearance of 0.58–2.63 L/h/kg, and an elimination half-life of ~5 h [43]. Liver metabolism represents the major clearance route (~90%), mainly involving CYP2D6, CYP2C19, and CYP3A4. In our massively intoxicated ECMO-treated case, without information on the ingested venlafaxine dose, we could only estimate the elimination half-life, found at 4-fold the reported values at a therapeutic dose of the extended released venlafaxine (5 ± 2 h). This clearly suggests a saturation of liver metabolism as confirmed by the non-linear *O*-desmethyl-venlafaxine concentration-versus-time curve and the parallelism of terminal slopes between venlafaxine and its *N*-desmethyl metabolite, indicating formation-dependent kinetics. ECMO allowed the maintaining of organ perfusion across 8 days until marked venlafaxine clearance and consequent cardiac failure reversal. We also confirmed that *O*-desmethyl-venlafaxine was the main venlafaxine metabolite both at toxic and therapeutic concentrations with, in our case, a metabolic ratio of 0.3 versus 0.06 for *N*-desmethyl-venlafaxine.

Using an untargeted approach based on LC-HRMS, we identified sixteen venlafaxine metabolites, including nine phase-I metabolites and seven phase-II metabolites, and determined their overall impact on the endometabolome. The *O*-, *N*-desmethyl, *N*,*O*-didesmethyl, and *N*,*N*,*O*-tridesmethyl-metabolites of venlafaxine and the *O*-desmethyl- and *N*,*O*-didesmethyl-venlafaxine-glucuronides have been previously described in human plasma [8]. Eleven additional metabolites were annotated. Based on data acquired in the positive ion mode, an MN including venlafaxine and all phase-I/phase-II metabolites were generated as previously described [37]. Clusterization was based on the common product ions displayed by all MS/MS spectra of venlafaxine metabolites, including the methoxyphenyl and hydroxycyclohexyl radical. Of note, clusterization was strengthened by the MS/MS spectra of eight *O*-desmethyl metabolites displaying transitions between precursors and several product ions involved in the networking. For instance, venlafaxine, for which MS/MS spectra displayed a transition (278.2114 > 121.0651), was clustered with *O*-desmethyl-venlafaxine, for which MS/MS spectra displayed a transition (264.1959 > 107.0496). The annotation reliability of venlafaxine metabolites was supported by the retention time, the exact mass measurement, and the manual inspection of tandem mass spectra. Note that the MS/MS spectra of annotated venlafaxine metabolites were uploaded in the GNPS library to be fully accessed by other researchers.

Among the sixteen venlafaxine metabolites, we have putatively annotated a hexosylated metabolite. Although not commonly reported in drug metabolism, hexosylation has been previously described with diphenhydramine, supported by the chemical synthesis of diphenidramine-*N*-glucose [44]. Here, no synthesis of venlafaxine-hexose was performed to confirm metabolite annotation, thus classified as being level-2 based on the metabolomic standard initiative guidelines [45]. To the best of our knowledge, hexosyl of venlafaxine has been identified for the first time, supporting possible venlafaxine metabolism via gut microbiota, known to be responsible for hexosyl metabolization [46]. Parallel kinetic profiles between venlafaxine and venlafaxine-hexose showed no delay between the metabolite and parent drug suggesting no first-pass effect. Nevertheless, additional studies are still needed to clarify hexosylation pathways.

Investigating the kinetics of annotated metabolites with no available absolute quantification provides useful information. We checked whether a semi-quantification procedure could provide sufficient accuracy to establish a kinetic profile. Plasma venlafaxine concentrations obtained via absolute and semi-quantitative procedures were correlated. This correlation appeared to be excellent, suggesting the accuracy of the semi-quantification-based approach to estimate plasma concentrations of venlafaxine metabolites lacking analytical standards. We consequently established the kinetic profile of 14 phase-I/phase-II venlafaxine metabolites. As expected, phase-II metabolites peaked later after their parent phase-I metabolites. The time-course of *O*-desmethyl-venlafaxine and *N*,*O*-didesmethyl-venlafaxine exhibited a plateau between Days 4 and 12, indicating a saturation of their metabolism, while their corresponding glucurono- and sulfo-conjugated metabolites regularly increased and peaked at the end of the plateau. The subsequent decrease in phase-I/phase-II metabolites coincided, likely related to improved hemodynamic conditions and a reduced volume of distribution with ECMO discontinuation. Of note, the plasma concentrations of glucurono-metabolites of *O*-desmethyl-venlafaxine and *N*,*O*-didesmethyl-venlafaxine on Day 24 were still higher than the initial samples (~140% and 215%, respectively). On Day 24, concentrations of sulfo-conjugated metabolites of *O*-desmethyl-venlafaxine and *N*,*O*-didesmethyl-sulfo-conjugated metabolites increased by 79% and 1230% in comparison to the initial samples, respectively. In contrast, venlafaxine and phase-I metabolites only represented 0.02% and ~2% of the initial plasma concentrations, respectively. Thus, assessing the kinetics of phase-II metabolites appeared to be relevant in determining drug detection windows for screening tests. Assessing multiple metabolic ratios between phase-II and I metabolites also provided insights toward identifying the delay between drug ingestion and blood sampling. However, additional studies with controlled conditions of drug ingestion are still required before definitive conclusions can be drawn.

Cardiovascular failure resulting from massive venlafaxine ingestion may have markedly altered liver metabolism. Besides the extensive characterization of venlafaxine metabolites, our analytical workflow assessed endogenous metabolite profiles. Here, ~210 endogenous metabolites, representative of what could be expected from metabolomics, were detected and annotated from plasma extracts including organic acids, amino acids, amino acid derivatives, and lipids. Estimated plasma concentrations and correlations to plasma venlafaxine were obtained from the 26 samples using the normalized intensity approach described for venlafaxine metabolites. Strong correlations were observed with 127 metabolites including 32 amino acids and derivatives, mainly essential amino acids such as tryptophan and excitatory neurotransmitters such as glutamic acid. While essential amino acids were positively correlated with plasma venlafaxine, glutamic acid was negatively correlated. Similar trends were observed in a ^1^H NMR-based metabolomic study performed on astrocytes in vitro, showing a decrease in intracellular glutamic acid concentrations following incubation with venlafaxine [47]. Interestingly, glutamic acid may represent a target of antidepressant drugs since altered concentrations have been reported in depression disorder patients [32,33,48]. Our findings have suggested that venlafaxine toxicity, at least in part, could be attributed to the inhibition of excitatory amino acids and/or to the dysregulation of amino acid homeostasis.

Plasma tryptophan concentrations were also correlated with plasma venlafaxine concentrations. This amino acid is known as the precursor of serotonin, a key venlafaxine target. Serotonin was only detected in the very first plasma samples, as expected with dose- and time-dependent decreasing concentrations [49,50]. Following biosynthesis, serotonin is mainly sequestered in platelets while plasma concentration only accounts for 5–10% of total blood serotonin [49,50]. Tryptophan catabolism depends on serotonin but also involves the kynurenine pathway. In our study, plasma concentrations of kynurenine, anthranilic acid, and nicotinamide, three metabolites of the kynurenine pathway, were positively correlated with plasma venlafaxine concentration. The promotion of the kynurenine pathway has been reported to result in the inhibition of serotonin synthesis [51], thus strongly supporting the notion that venlafaxine may decrease plasma serotonin concentrations by activating the kynurenine pathway. Some metabolites in the kynurenine pathway, including kynurenic acid, have been described as being neuroprotective in the depression setting [52,53,54,55,56]. Interestingly, kynurenic acid was the only negatively correlated metabolite with venlafaxine. Glutamic acid, known to be modulated by kynurenic acid, was also the only amino acid to be negatively correlated with plasma venlafaxine concentration. Studies have indicated that the kynurenine pathway might be activated in cardiotoxicity mechanisms, thus suggesting a contribution of homeostasis disruption of kynurenine metabolites to venlafaxine-mediated toxicity [57,58,59].

The first available plasma sample showed the lowest lipid content, which increased regularly and markedly over the time as indicated by the negative correlation of phosphatidylcholine, phosphatidylethanolamine, phosphatidylinositol, and their corresponding lysophospholipid subclasses with venlafaxine concentration. The fatty acyl side chain of phospholipids was various, including stearic, palmitoic, arachidonic, and docohexaenoic acid. To the best of our knowledge, such a change in plasma lipid homeostasis has not been reported in venlafaxine poisoning. We hypothesized that the dramatic decrease in plasma lipid concentration observed in the initial samples was related to increased needs in β-oxidation-mediated energy production, especially by the stressed poisoned heart.

Our study has limitations despite very prolonged plasma sampling over 25 days. First of all, this study has been performed on a single patient and thus does not take into account interindividual variability which affects the metabolic pattern, especially since venlafaxine is metabolized by CYP enzymes, displaying a large phenotypical variability. Our observations of the homeostasis disruption of endogenous metabolome thus require complementary investigations. Cardiac failure and ECMO implementation may have been responsible for a part of the observed metabolic changes, including the homeostasis disruption of amino acids, the Krebs cycle, and the kynurenine pathway. Our study could not differentiate the specific metabolic consequences of venlafaxine poisoning from those related to cardiovascular complication and its therapies. However, our study is the first toxicometabolomic approach in clinical toxicology and could be considered as a first step towards personalized metabolomics for clinical management. The metabolomic approach seems suitable for identifying metabolic outcomes in severe poisonings and for raising mechanistic hypotheses before confirmation using in vitro or in vivo models. Studies are needed to investigate whether such identified metabolic alterations might be considered as biomarkers predictive of severity or ECMO requirement in venlafaxine or membrane-stabilizing agent poisoning. Finally, since oxazepam found in toxic concentrations in our case is also a uridine 5’-diphospho-glucuronosyltransferase substrate, we could not rule out competition at glucuroconjugation with venlafaxine metabolites.

## 6. Conclusions

We performed a complete metabolomic investigation in a severely venlafaxine-poisoned patient. Targeted analyses allowed for quantifying and investigating the pharmacokinetics of venlafaxine and its demethylated metabolites. Non-targeted analyses allowed for identifying other venlafaxine metabolites in a time-course manner providing new insights to extend the detection window of venlafaxine. Our approach based on correlations suggested that venlafaxine poisoning is responsible for major alterations in endogenous metabolites involved in Krebs cycle, amino acid, and lipid metabolisms. Although additional studies are needed to investigate whether these altered metabolites could be regarded as prognosticators of membrane-stabilizing agent poisoning, our study provides new insights into the mechanisms of venlafaxine-related toxicity and opens exciting perspectives in toxicometabolomics applied to clinical toxicology.

## Data Availability

Data presented in this article are available within this article and in Supplementary Data Table S2.

## Supplementary Materials

The following supporting information can be downloaded at https://www.mdpi.com/article/10.3390/metabo13030353/s1: Figure S1: Total ion chromatogram of QC 1/1 in positive and negative ion mode; Figure S2: Proposed fragmentation patterns of venlafaxine based on the inspection of the MS/MS spectra obtained through the analysis of the commercial standard; Figure S3: Isomers of hydroxylated venlafaxine metabolites. Figure S4: Metabolic network of venlafaxine and its phase-I and phase-II metabolites; Table S1: Analytical features of venlafaxine and its annotated metabolites; Table S2: Correlation between annotated endogenous plasma metabolites and venlafaxine concentrations.
