# Supplementary material for: Life-Threatening Cardiogenic Shock Related to Venlafaxine Poisoning—A Case Report with Metabolomic Approach
Source: Metabolites. 2023 Feb 27;13(3):353. doi: 10.3390/metabo13030353 (PMC10053335; doi:10.3390/metabo13030353)
Supplement: Supplementary file 1 [file metabolites-13-00353-s001.zip › Supporting_Informations.pdf]

**Supporting Informations.**

# Life-Threatening Cardiogenic Shock Related to Venlafaxine Poisoning—A Case Report with Metabolomic Approach

**Romain Magny [1,2,*], Bruno Mégarbane [2,3,*], Pauline Guillaud [1], Lucie Chevillard [2], Nicolas Auzeil [4], Pauline Thiebot [1,2], Sebastian Voicu [2,3], Isabelle Malissin [2,3], Nicolas Deye [3], Laurence Labat [1,2] and Pascal Houzé [1,5]**

1. Laboratoire de Toxicologie, Fédération de Toxicologie, AH-HP, Hôpital Lariboisière, 75010 Paris, France
2. Inserm, UMRS-1144, Université Paris Cité, 75006 Paris, France
3. Réanimation Médicale et Toxicologique, Fédération de Toxicologie de l'AH-HP, Hôpital Lariboisière, 75010 Paris, France
4. Université Paris Cité, CNRS, CiTCoM, 75006 Paris, France
5. Université Paris Cité, Faculté de Sciences Pharmaceutiques et Biologique, Unité de Technologies Chimiques et Biologiques pour la Santé (UTCBS), CNRS UMR8258, Inserm UMR-8258, 75006 Paris, France

* Correspondence: romain.magny@aphp.fr (R.M.); bruno.megarbane@lrb.aphp.fr (B.M.)

**Figure S1. Total ion chromatogram of QC 1/1 in (A) positive and (B) negative ion mode.**

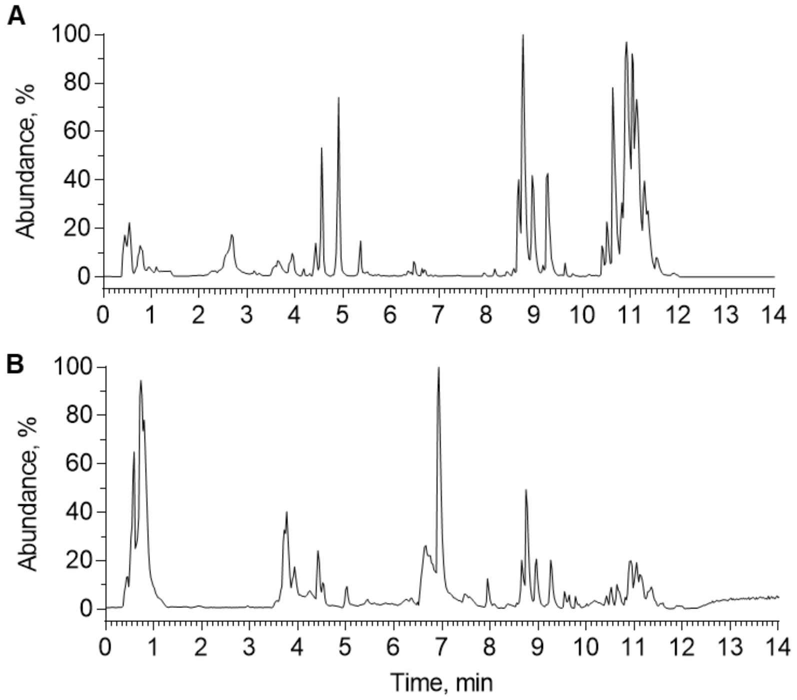

**Figure S2. Proposed fragmentation patterns of venlafaxine based on the inspection of the MS/MS spectra obtained through the analysis of the commercial standard.**

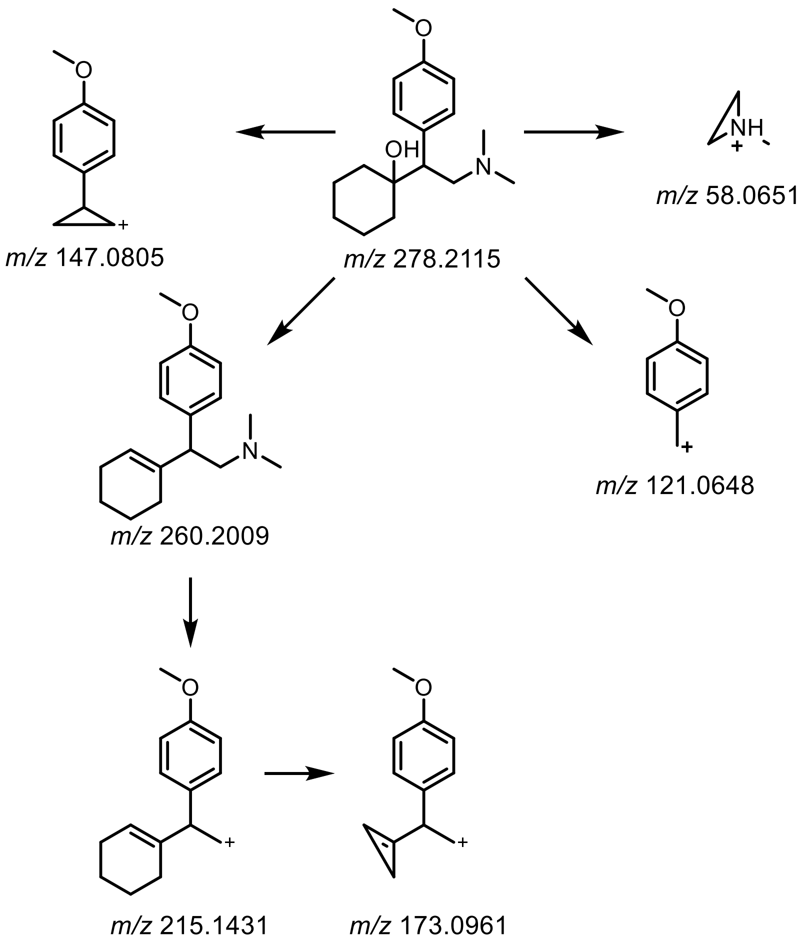

**Figure S3. Isomers of hydroxylated venlafaxine metabolites.** Extracted ion chromatogram at m/z 294.2064 corresponding to the [M+H]+ ion of hydroxyl venlafaxine.

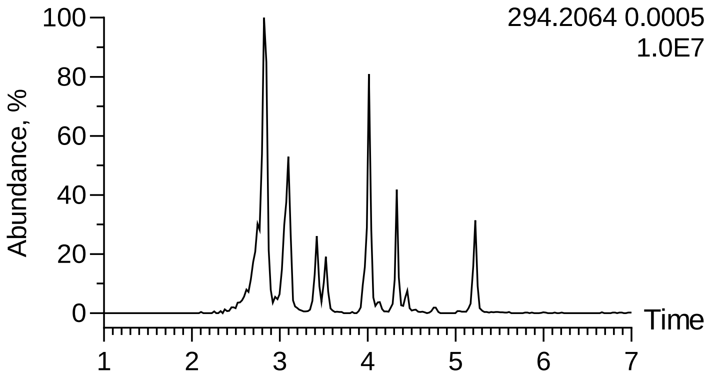

**Figure S4. Metabolic network of venlafaxine and its phase I and II metabolites.**

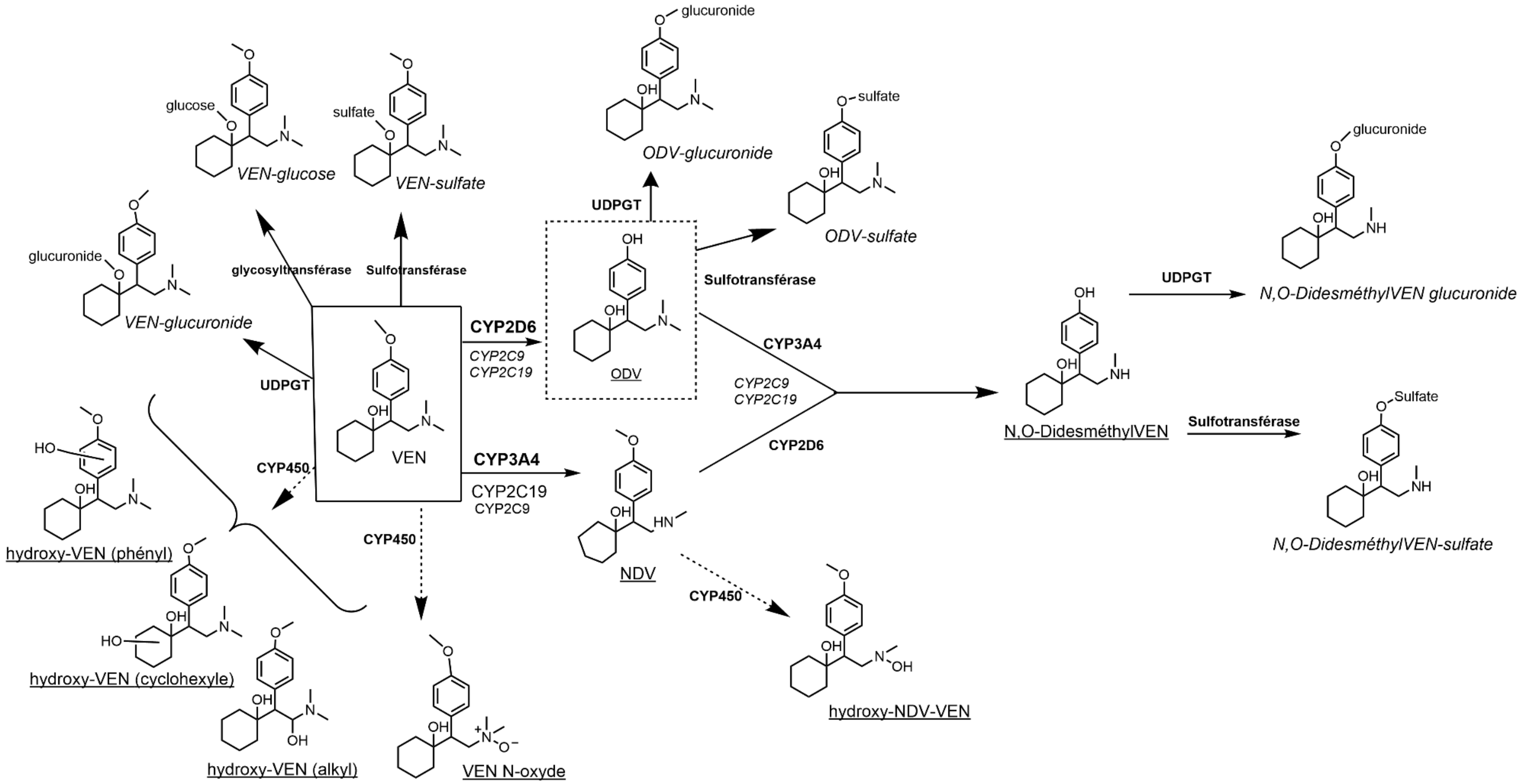

**Table S1. Analytical features of venlafaxine and its annotated metabolites.**

| Name | $t_R$ | Precursor ion *m/z* | Formula | Product ions *m/z* |
|---|---|---|---|---|
| Venlafaxine | 4.90 | 278.2115 | $C_{17}H_{27}NO_2$ | 58.0660, 121.0649, 173,0962, 215.1431, 260.2009 |
| O-desmethyl-venlafaxine | 3.96 | 264.1958 | $C_{16}H_{25}NO_2$ | 58.0661, 84.0452, 107.0496, 133.0651, 159,0805, 201.1277, 246,1850 |
| N-desmethyl-venlafaxine | 4.87 | 264.1958 | $C_{16}H_{25}NO_2$ | 121.0651, 147.0806, 173,0963, 215.1432, 246,1854 |
| N,O-Didesmethyl-Venlafaxine | 3.9 | 250.1801 | $C_{15}H_{23}NO_2$ | 107.0496, 133.0649, 159,0804, 201.1267, 232.1690 |
| N,N,O-tridesmethyl-Venlafaxine | 3.8 | 236.1645 | $C_{14}H_{21}NO_2$ | 107.0497, 133.0651, 159,0808, 201.1271, 232.1694 |
| Venlafaxine N-Oxide | 5.22 | 294.2062 | $C_{17}H_{27}NO_3$ | 58.0660, 121.0651, 215.1440 |
| OH-Venlafaxine - Alkyl | 4.01 | 294.2064 | $C_{17}H_{27}NO_3$ | 58.0661, 121;0652, 171.0806, 213.1271, 276.1962 |
| OH-Venlafaxine -Cyclohexyl | 2.84 | 294.2062 | $C_{17}H_{27}NO_3$ | 58.0661, 121.0650, 171,0805, 213,1278, 276,1962 |
| OH-Venlafaxine - Phenyl | 4.33 | 294.2063 | $C_{17}H_{27}NO_3$ | 58.0661, 107.0862, 137,0599, 199.1118, 278.1953 |
| O-Desmethyl-Venlafaxine-N-Oxide | 4.25 | 280.1906 | $C_{16}H_{25}NO_3$ | 107.0496 |
| N-Desmethyl-Hydroxy-Venlafaxine | 2.92 | 280.1906 | $C_{16}H_{25}NO_3$ | 84.0451, 121.0651, 171.0799, 262.1804 |
| Venlafaxine Hexose | 4.91 | 440.2639 | $C_{23}H_{37}NO_7$ | 58.0661, 121.0651, 147.0805, 173.0957, 260.2007, 278.2114 |
| Venlafaxine Glucuronide | 5.02 | 454.2431 | $C_{23}H_{35}NO_8$ | 58.0661, 121.0651, 135.0805, 147.0806, 173.0957, 215.1432, 260.2006, 278.2112 |
| O-desmethyl-Venlafaxine-Glucuronide | 3.25 | 440.2275 | $C_{22}H_{33}NO_8$ | 58.0660, 107.0496, 133.0649, 173.0966, 201.1273, 246.1847, 264.1951 |
| O-desmethyl Venlafaxine-sulfate | 4.23 | 344.1536 | $C_{16}H_{25}NO_5S$ | 58.0661, 107.0496, 133.0651, 159.0805, 192.1019, 206.1179, 246.1851 |
| N,O-Didesmethyl-Venlafaxine-Glucuronide | 3.17 | 426.2118 | $C_{21}H_{31}NO_8$ | 137.0598, 151.0755, 178.0863, 192.1020 |
| N,O-Didesmethyl-Venlafaxine-Sulfate | 4.2 | 328.1228 | $C_{15}H_{23}NO_5S$ | 137.0597, 151.0757, 178.0865, 192.1025 |
